# From Glucosinolate Content to Isothiocyanate Yield: Rethinking *Brassica* Functionality

**DOI:** 10.3390/nu18162624

**Published:** 2026-08-11

**Authors:** Anna Wondołowska-Grabowska, Ewa Szpunar-Krok, Michał Węgielewski, Monika Kowalska-Góralska, Magdalena Senze, Maria Czernicka

**Affiliations:** 1 Institute of Agroecology and Plant Production, Wrocław University of Environmental and Life Sciences, Grunwaldzki Sq. 24A, 50-363 Wrocław, Poland; michal.wegielewski@wp.pl; 2 Department of Crop Production, Faculty of Technology and Life Sciences, University of Rzeszów, Zelwerowicza 4 St., 35-601 Rzeszow, Poland; eszpunar@ur.edu.pl; 3 Department of Limnology and Fishery, Institute of Animal Breeding, Wroclaw University of Environmental and Life Sciences, ul. Chełmońskiego 38c, 51-630 Wrocław, Poland; monika.kowalska-goralska@upwr.edu.pl (M.K.-G.); magdalena.senze@upwr.edu.pl (M.S.); 4 Department of Bioenergetics, Food Analysis and Microbiology, Faculty of Technology and Life Sciences, University of Rzeszów, Zelwerowicza 8b St., 35-601 Rzeszow, Poland; mczernicka@ur.edu.pl

**Keywords:** *Brassica* vegetables, glucosinolates, isothiocyanates, ITC yield, myrosinase, conversion efficiency, food processing

## Abstract

**Background/Objectives**: *Brassica* vegetables are widely promoted as health-promoting foods, but their functional value is still commonly inferred from glucosinolate content alone. However, glucosinolates are biologically inactive precursors whose nutritional relevance depends on their conversion into bioactive hydrolysis products, particularly isothiocyanates (ITCs). This review aims to reconsider *Brassica* functionality through an ITC-yield-centered perspective rather than a precursor-content-based approach. **Methods**: This narrative review synthesizes current evidence on the biochemical, technological, gastrointestinal, and analytical determinants of glucosinolate hydrolysis and ITC formation. Particular attention is given to myrosinase activity, specifier proteins, pH, temperature, tissue disruption, food matrix effects, processing conditions, gastrointestinal transformation, inter-individual microbial variability, and the limitations of precursor-based interpretation. **Results**: The reviewed evidence indicates that similar glucosinolate profiles can result in markedly different levels of bioactive exposure depending on enzymatic activity, processing history, matrix context, and host-related factors. Therefore, precursor abundance alone is an unreliable surrogate for functional efficacy. Functional optimization should not aim at indiscriminate maximization of glucosinolate breakdown, but rather at selective promotion of beneficial ITC-forming pathways while limiting nitriles, epithionitriles, and goitrogenic products such as goitrin. Conversion efficiency emerges as a critical analytical bridge between glucosinolate abundance and food-level ITC formation, while subsequent bioaccessibility and host-related factors determine realized exposure. **Conclusions**: *Brassica* functionality could be more meaningfully evaluated through an ITC-yield-centered framework in which precursor abundance and conversion efficiency determine food-level ITC formation, while subsequent bioaccessibility and host-related factors shape realized exposure. This approach provides an integrative basis for breeding, food processing, product development, dietary guidance, and future evaluation of *Brassica* foods.

## 1. Introduction: Why Glucosinolate Content Is No Longer Enough

*Brassica* vegetables, including broccoli, cabbage, kale, Brussels sprouts, cauliflower, and related edible crops, are important components of plant-based diets and are widely recognized for their potential contribution to chronic disease prevention. Their regular consumption has been associated with reduced cancer risk, cardiovascular protection, and anti-inflammatory effects, which have traditionally been attributed primarily to their glucosinolate content [1,2,3]. In nutritional, agronomic, and food-quality research, glucosinolate concentration has therefore often been used as a convenient proxy for the health-promoting potential of *Brassica* foods. Breeding programs, agricultural practices, and dietary recommendations have similarly tended to emphasize the selection or promotion of materials with high glucosinolate abundance [4].

However, this precursor-based interpretation has important limitations. Glucosinolates themselves are not the main bioactive endpoints responsible for many of the biological effects attributed to *Brassica* vegetables. Rather, they function as relatively stable precursors whose nutritional relevance depends on their conversion into hydrolysis products, particularly isothiocyanates (ITCs), through myrosinase-catalyzed hydrolysis [5]. This conversion is highly conditional rather than automatic. Myrosinase activity is sensitive to thermal inactivation, pH changes, and the extent of tissue disruption, all of which can substantially alter the formation of ITCs from the same glucosinolate pool [6,7,8,9]. In addition, competing hydrolysis pathways mediated by epithiospecifier proteins (ESPs) can redirect glucosinolate degradation toward nitriles or other alternative products instead of ITCs, thereby reducing the expected functional output [7,10]. Food preparation and processing further complicate this relationship. Domestic and industrial treatments such as boiling, steaming, microwaving, stir-frying, fermentation, and high-pressure processing can markedly modify glucosinolate retention, myrosinase activity, specifier-protein activity, and final ITC yield, in ways that are not always predictable from precursor abundance alone [11,12]. Moreover, *Brassica* foods are rarely consumed as isolated plant tissues. Their functional output may be influenced by the broader food matrix, including co-ingested ingredients, pH modifiers, lipid and protein interactions, and the use of exogenous enzyme sources capable of restoring or enhancing hydrolysis after processing [13,14,15]. These factors indicate that the biological relevance of *Brassica* foods depends not only on what is present in the raw plant, but also on what is actually formed, preserved, released, absorbed, and metabolized after preparation and consumption.

Despite this complexity, glucosinolate content continues to be frequently reported and interpreted as the principal indicator of *Brassica* functionality, whereas ITC yield and conversion efficiency are less consistently considered [2,16]. This creates a critical interpretative gap: foods with similar glucosinolate profiles may generate markedly different levels of bioactive ITC exposure depending on cultivar, tissue type, processing conditions, preparation practices, gastrointestinal transformation, and inter-individual microbial variability [17,18,19,20]. As a result, dietary recommendations, food formulations, and intervention studies based only on glucosinolate abundance may overestimate or underestimate the actual bioactive potential of *Brassica* foods, particularly when ITC bioavailability is limited or unpredictable [15,21].

For clarity, the present review distinguishes several sequential but non-equivalent concepts. Conversion efficiency refers to the proportion of an available glucosinolate pool that is converted into isothiocyanates under defined conditions, whereas ITC yield denotes the amount of ITCs actually formed at the food or processing level. Bioaccessibility describes the fraction released from the food matrix and potentially available for intestinal absorption, while bioavailability and realized exposure refer to the fraction that is absorbed and subsequently reflected in systemic or excreted metabolites. Biological effectiveness represents a further downstream level and depends not only on exposure but also on dose, host metabolism, target-tissue responsiveness, and duration of exposure. These distinctions are essential because efficient ITC formation in a food does not necessarily translate proportionally into systemic exposure or biological effect.

Previous reviews have comprehensively addressed glucosinolate biosynthesis and distribution, the effects of processing and digestion on ITC formation, and the human metabolism and bioavailability of glucosinolate-derived products [2,7,16]. Building on this foundation, the present review integrates these areas within an ITC-yield-centered interpretative framework that organizes precursor abundance, conversion efficiency, food-level ITC formation, digestive bioaccessibility, systemic exposure, and biological effectiveness as sequential but non-equivalent levels of evidence. By positioning conversion efficiency as the analytical bridge between glucosinolate composition and actual ITC formation, the review examines why precursor abundance alone cannot reliably predict bioactive delivery and links plant-, processing-, matrix-, gastrointestinal-, host-, and analytical determinants within a common conceptual sequence. This perspective provides a more explicit basis for evaluating when glucosinolate-rich *Brassica* foods are likely to translate into meaningful bioactive exposure and has direct implications for breeding, food processing, product formulation, dietary guidance, and the design of future intervention studies.

## 2. Literature Search Strategy

This narrative review was supported by a structured literature search designed to identify studies directly relevant to glucosinolate hydrolysis, isothiocyanate formation, and the factors determining their functional and nutritional interpretation in *Brassica* foods. Literature was identified using PubMed and Google Scholar, supplemented by backward and forward citation tracking of key publications. The search primarily covered studies published from 2009 to July 2026, with emphasis on recent evidence from 2015–2026 while retaining earlier foundational studies where necessary to establish key biochemical or analytical mechanisms. Search terms were used in different combinations and included “*Brassica*”, “*Broccoli*”, “*Cabbage*”, “*Kale*”, “*Cauliflower*”, “mustard”, “glucosinolate”, “isothiocyanate”, “sulforaphane”, “glucoraphanin”, “myrosinase”, “epithiospecifier protein”, “ESP”, “glucosinolate hydrolysis”, “conversion efficiency”, “ITC yield”, “food processing”, “cooking”, “fermentation”, “high-pressure processing”, “food matrix”, “bioaccessibility”, “bioavailability”, “gut microbiota”, “urinary biomarkers”, “human intervention”, and “isothiocyanate quantification”. Additional targeted searches were conducted when specific mechanistic, analytical, processing-related, or human-exposure questions emerged during manuscript development.

Priority was given to peer-reviewed original studies directly examining glucosinolate retention and hydrolysis, myrosinase or specifier-protein activity, ITC formation, food-level conversion, processing and matrix effects, gastrointestinal transformation, bioaccessibility, bioavailability, human exposure, and analytical quantification. High-quality reviews were used primarily to identify relevant primary studies, provide mechanistic context, and trace earlier foundational evidence. Studies involving model or non-food *Brassicaceae* were retained only when they provided mechanistic information directly applicable to the glucosinolate–myrosinase system. Duplicate records, conference abstracts, non-peer-reviewed sources, and studies without a direct connection to glucosinolate-to-ITC conversion or its nutritional interpretation were excluded. Because the review was designed as a critical narrative synthesis rather than a systematic review or meta-analysis, study selection was guided by thematic relevance and evidential contribution rather than by a formal risk-of-bias scoring procedure.

## 3. Glucosinolates as Precursors, Not Endpoints

Glucosinolates are β-thioglucoside *N*-hydroxysulfates with a variable side chain (R group) that determines their classification as aliphatic, aromatic, or indole glucosinolates. Representative examples include glucoraphanin and sinigrin among aliphatic glucosinolates, gluconasturtiin among aromatic glucosinolates, and glucobrassicin among indole glucosinolates [22]. More than 130 glucosinolates have been identified, with their distribution varying according to species, cultivar, tissue type, and developmental stage [23,24]. Glucoraphanin (4-methylsulfinylbutyl glucosinolate) in broccoli and sinigrin (2-propenyl glucosinolate) in mustard are among the most extensively studied compounds because of their high ITC-forming potential [3,25].

Upon tissue disruption, glucosinolates come into contact with myrosinase (thioglucoside glucohydrolase, EC 3.2.1.147), a β-thioglucosidase that initiates glucosinolate breakdown through enzymatic reactions further shaped by associated degradation enzymes and specifier proteins [26,27]. The resulting unstable aglycone may spontaneously rearrange to form ITCs, nitriles, thiocyanates, or epithionitriles, depending on pH, temperature, and the presence of specifier proteins [5,28]. At neutral to slightly acidic pH values, approximately 5.0–7.0, and in the absence of active ESPs, ITCs are generally the predominant products [6]. By contrast, at pH < 3.5 or in the presence of active ESPs, nitrile formation is favored, thereby reducing ITC yield [29,30]. ITCs, particularly sulforaphane derived from glucoraphanin and allyl isothiocyanate derived from sinigrin, exhibit a broad range of biological activities, including induction of phase II detoxification enzymes via Nrf2 activation, inhibition of phase I enzymes, modulation of inflammatory pathways, antimicrobial effects, and epigenetic regulation [31,32]. Nitriles, by contrast, generally lack these activities and may raise safety concerns at high exposure levels [33]. Thus, the biological functionality of *Brassica* vegetables is not determined by glucosinolate content per se, but by the efficiency and direction of conversion toward bioactive ITCs, a process governed by enzymatic, physicochemical, and contextual factors.

Importantly, not all glucosinolate-derived products should be viewed as desirable endpoints of functional optimization. While ITCs such as sulforaphane and allyl isothiocyanate are widely associated with chemoprotective and anti-inflammatory activity, other hydrolysis products may be biologically neutral, less beneficial, or undesirable from a nutritional perspective [2,31]. In addition to nitriles and epithionitriles, certain glucosinolates such as progoitrin may yield goitrin upon hydrolysis, a compound associated with goitrogenic activity and antinutritional relevance, particularly in the context of high exposure or compromised iodine status [34,35]. This distinction is also relevant from a food-processing perspective, because some culinary strategies may aim not only to preserve ITCs but also to reduce goitrin formation, highlighting that different hydrolysis products do not contribute equally to functional value [36]. Accordingly, the functional objective is not the indiscriminate maximization of glucosinolate conversion, but the selective promotion of beneficial ITC-forming pathways while minimizing the formation of undesirable or potentially harmful products. This distinction is essential for interpreting *Brassica* functionality in a biologically meaningful and nutritionally responsible way [2,35].

## 4. Glucosinolate Hydrolysis as a Functional Bottleneck

**Figure 1:**
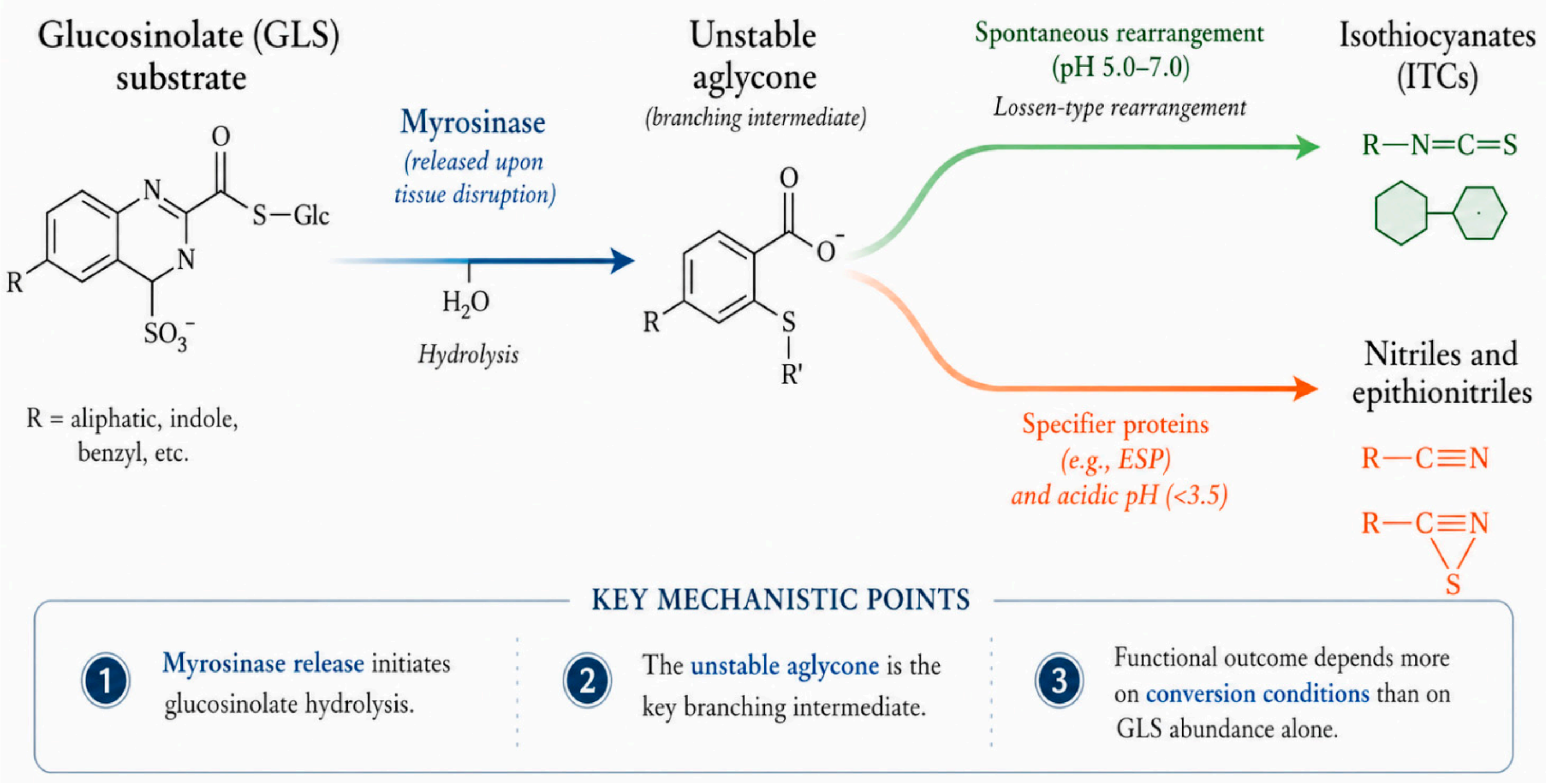
The glucosinolate–myrosinase bottleneck determining whether glucosinolate hydrolysis yields bioactive isothiocyanates or less functional nitriles and epithionitriles. The scheme emphasizes the unstable aglycone as the key branching intermediate and highlights that conversion conditions, rather than glucosinolate content alone, govern functional outcome. Blue denotes myrosinase-mediated hydrolysis, green denotes the pathway leading to isothiocyanates, and orange denotes the alternative formation of nitriles and epithionitriles.

To clarify the mechanistic branching point that determines whether glucosinolate hydrolysis yields bioactive isothiocyanates or less functional alternative products, the core glucosinolate–myrosinase bottleneck is summarized in Figure 1.

As shown in Figure 1, functional outcome depends not simply on precursor abundance, but on whether hydrolysis proceeds under conditions favoring isothiocyanate formation or is redirected toward nitrile-producing pathways. This mechanistic bottleneck is particularly relevant from a nutritional perspective because it determines whether a *Brassica* food with a given glucosinolate profile actually delivers bioactive ITCs after preparation and consumption. Section Enzymatic, Structural, and Tissue-Level Determinants of Glucosinolate Hydrolysis examines this bottleneck in terms of myrosinase activity, ESP-mediated diversion, glucosinolate-specific hydrolysis behavior, and tissue compartmentalization.

### Enzymatic, Structural, and Tissue-Level Determinants of Glucosinolate Hydrolysis

Myrosinase is the principal enzymatic determinant of ITC formation, but its activity varies across species, cultivars, tissues, and developmental stages [4,37]. Broccoli florets generally exhibit higher activity than stems, while young leaves of leafy *Brassica* vegetables show greater activity than mature leaves [37]. Its catalytic performance is strongly influenced by temperature, pH, substrate concentration, and tissue disruption, making myrosinase activity highly context-dependent [6,9]. Boiling for 5–10 min can reduce myrosinase activity by more than 90% [11,17], while fermentation-associated acidification can strongly influence ITC formation, making careful control of fermentation conditions important [38,39,40]. The broader processing implications of these dependencies are discussed in Section 5. Importantly, myrosinase activity and glucosinolate-to-ITC conversion efficiency represent related but distinct analytical concepts. Enzyme activity or kinetic parameters describe the catalytic performance of myrosinase under defined assay conditions and are influenced by substrate concentration, temperature, pH, and enzyme stability [6,22]. Conversion efficiency, in contrast, is a system-level outcome describing the proportion of an available glucosinolate pool ultimately converted into the corresponding ITC. It therefore reflects not only myrosinase activity, but also substrate accessibility, tissue disruption, competition from specifier-protein-mediated pathways, product instability, processing conditions, and matrix effects [6,30,33]. Consequently, high myrosinase activity does not necessarily translate proportionally into high ITC conversion efficiency.

A further determinant is the activity of epithiospecifier proteins (ESPs), non-heme iron proteins that redirect glucosinolate hydrolysis toward nitriles, epithionitriles, and thiocyanates rather than ITCs [5,30,41,42]. Their activity is tissue- and development-dependent, with higher levels reported in roots and seeds than in leaves and florets [30,43,44]. In kohlrabi, ESP activity in the peel may be 3–5 times higher than in the flesh, producing markedly different ITC/nitrile ratios despite similar glucosinolate content [30,45]. ESP-mediated diversion is also influenced by pH and temperature: acidic conditions favor nitrile formation, whereas higher pH promotes ITC formation [6,29]. Because ESPs are generally more heat-stable than myrosinase, mild heating may reduce myrosinase activity before fully inactivating ESPs [45], unintentionally favoring nitrile formation and limiting ITC yield [29].

Glucosinolate structure further influences hydrolysis kinetics and product distribution. Not all glucosinolates hydrolyze at the same rate, and recent work suggests that myrosinase-mediated degradation pathways may vary in greater mechanistic detail than previously assumed, even within broccoli-based systems [46,47]. Aliphatic glucosinolates, such as glucoraphanin and sinigrin, are generally hydrolyzed more rapidly than aromatic or indole glucosinolates [46], while side-chain length and functional groups also affect hydrolysis behavior [24,48]. Glucoraphanin, with a four-carbon aliphatic side chain and a methylsulfinyl group, is efficiently converted to sulforaphane under favorable conditions, whereas glucobrassicin, an indole glucosinolate, yields indole-3-carbinol and related products with distinct biological activities [2,16].

Tissue compartmentalization adds another level of control. Glucosinolates are stored in vacuoles, while myrosinase resides in myrosin cells or idioblasts [49]. Effective hydrolysis therefore requires tissue disruption to bring enzyme and substrate into contact [7]. Inadequate chewing, minimal processing, or consumption of intact tissues can consequently result in low ITC yield despite high glucosinolate content [14,18].

## 5. Food Processing as a Determinant of Isothiocyanate Yield

### 5.1. Thermal Processing: A Double-Edged Sword

Thermal processing is the most common intervention in *Brassica* preparation, yet its effects on ITC yield are complex and often counterintuitive. Boiling is particularly detrimental: it inactivates myrosinase, leaches glucosinolates into cooking water, and results in near-zero ITC formation [6,11]. Steaming, by contrast, preserves myrosinase activity at short durations (3–5 min) and can maintain or even enhance ITC yield by facilitating tissue disruption and enzyme-substrate contact [12,14]. However, prolonged steaming (>10 min) inactivates myrosinase and reduces ITC yield [17,36].

Microwaving presents a similar trade-off. Short microwaving (1–2 min) can increase ITC yield by disrupting tissues and activating myrosinase, while prolonged microwaving (>3 min) inactivates the enzyme [11,50,51]. Stir-frying, a high-temperature, short-duration method, can preserve myrosinase activity if cooking times are brief (<3 min), but extended stir-frying reduces ITC yield [11,12].

A mechanistically justified strategy is the “hydrolysis-before-cooking” approach, whereby tissue disruption and myrosinase-mediated hydrolysis are allowed to proceed before thermal treatment [12,32]. For example, chopping broccoli and leaving it to stand for 30–90 min before cooking can increase ITC yield by 2–3-fold relative to immediate cooking, because ITCs formed during the pre-hydrolysis phase are more thermally stable than myrosinase itself [12,25]. By effectively decoupling ITC formation from subsequent heating, this strategy maximizes conversion efficiency while maintaining palatability. Because the available studies differ substantially in plant material, processing protocols, analytical endpoints, and experimental design, direct numerical comparison across studies remains difficult. Accordingly, Table 1 presents literature-derived ranges as illustrative estimates of the direction and approximate magnitude of processing effects on glucosinolate-to-isothiocyanate conversion, myrosinase activity, ESP activity, and related matrix-dependent factors rather than as universal quantitative thresholds.

### 5.2. Non-Thermal and Enzyme-Assisted Strategies

Non-thermal and enzyme-assisted strategies offer complementary approaches to improve ITC yield when conventional heating compromises endogenous myrosinase activity. High-pressure processing (HPP) is one of the most promising non-thermal technologies in this context. Pressures of 400–600 MPa can disrupt plant tissues and enhance enzyme–substrate contact without fully inactivating myrosinase, thereby supporting increased ITC yield [7,59]. However, pressures above 600 MPa may denature myrosinase and reduce ITC formation [59]. HPP may also inactivate ESPs at lower pressures than those required for myrosinase inactivation, potentially shifting glucosinolate hydrolysis toward ITC formation rather than nitrile production [66].

Fermentation, particularly lactic acid fermentation, represents another processing strategy with potential relevance for *Brassica*-based functional foods [67]. The effect of fermentation on ITC yield is strongly pH-dependent. Mild acidification, approximately pH 5.0–5.5, may support ITC formation by disrupting tissues, releasing glucosinolates, and maintaining partial myrosinase activity [38,39,68]. By contrast, excessive acidification below pH 4.5 can reduce myrosinase activity and favor nitrile formation, thereby limiting the expected functional output [40,69]. Fermentation with myrosinase-positive bacteria, such as *Lactobacillus plantarum*, may further enhance ITC yield by supplementing endogenous enzymatic activity, although this effect depends on strain selection, substrate composition, and fermentation conditions [40,70].

Enzyme-assisted strategies are particularly relevant when endogenous myrosinase has already been inactivated during processing. Supplementing cooked or processed *Brassica* foods with exogenous myrosinase from mustard seeds, daikon radish, or microbial sources can restore ITC formation from residual glucosinolates [3,13]. This approach is supported by evidence that mustard-derived myrosinases differ in thermal and pressure stability, which is important for their potential use as functional processing aids in formulated foods [60,71]. Okunade et al. [13] reported that supplementation with mustard seed-derived exogenous myrosinase increased sulforaphane bioavailability after cooked broccoli consumption in healthy subjects. More recently, Mastaloudis et al. [15] confirmed in a randomized clinical study that mustard seed myrosinase can enhance sulforaphane bioavailability from a glucoraphanin-rich broccoli seed extract. This strategy is therefore especially relevant for processed foods, ready-to-eat meals, and dietary supplements in which endogenous myrosinase is absent or inactive [21,25].

Overall, these approaches show that processing should not be interpreted only as a cause of glucosinolate loss or enzyme inactivation. When properly designed, non-thermal processing, controlled fermentation, and exogenous myrosinase supplementation can be used to improve the predictability of ITC formation and support the development of *Brassica*-based foods with more consistent nutritional functionality.

## 6. Matrix Effects and Food Design: *Brassica* Functionality Depends on the System, Not Only the Plant

To avoid conflating distinct levels of influence, matrix effects are considered here as physicochemical interactions arising from food composition and structure before and at ingestion, including co-formulated ingredients, local pH and buffering capacity, lipid partitioning, protein binding, and encapsulation. Digestive effects are treated separately in Section 7 and refer to transformations imposed by the gastrointestinal environment after ingestion, including gastric and intestinal pH, digestive release, microbial metabolism, absorption, and host processing [8,9,14,72,73,74]. Although these levels interact, a matrix effect on ITC formation or retention does not necessarily predict digestive bioaccessibility or systemic exposure.

### 6.1. Co-Ingested Foods and pH Modulation

The food matrix can influence food-level ITC formation and retention by modifying enzyme activity, substrate accessibility, hydrolysis direction, and compound stability. Because *Brassica* vegetables are usually consumed as components of mixed meals, co-formulated ingredients may alter local physicochemical conditions and enzyme–substrate interactions. However, these matrix-level effects should be distinguished from subsequent transformations imposed by the gastrointestinal environment. Available evidence suggests that processing conditions often exert a stronger influence than meal composition itself: in vitro, steaming time affected sulforaphane formation and bioaccessibility more strongly than protein or lipid addition, while a human intervention study similarly identified cooking duration as a more important determinant of sulforaphane yield than meal composition [14,75]. This suggests that intrinsic plant- and processing-related determinants frequently dominate over extrinsic meal factors, although matrix effects may differ depending on whether ITCs are formed in situ or ingested as preformed compounds.

Nevertheless, matrix effects remain relevant for the design of functional foods, ready-to-eat meals, and supplements in which *Brassica* ingredients are combined with other components. The behavior of glucosinolates and their hydrolysis products may differ in binary or multi-component food systems and formulated products, where co-ingredients can modify thermal stability, retention, and subsequent availability. Reduced glucosinolate degradation has been demonstrated in broccoli-based binary systems containing other food ingredients [70], while more recent evidence from broccoli-enriched bakery formulations also indicates that the surrounding food matrix can protect glucosinolates and other bioactive compounds during thermal processing [76].

### 6.2. Lipid and Protein Interactions

Lipids and proteins can further modify ITC stability, release, and bioaccessibility. Because many ITCs are relatively lipophilic, they may partition into lipid phases within the food matrix. This partitioning may support intestinal absorption in some contexts, but it can also reduce apparent aqueous bioaccessibility during digestion, depending on the structure and composition of the meal [72]. Therefore, lipid-containing formulations should be evaluated not only for their ability to retain ITCs, but also for their effects on release and availability under gastrointestinal conditions.

Proteins represent another important class of matrix components because ITCs are electrophilic and can react with nucleophilic amino acid residues. Such interactions may lead to the formation of protein–ITC adducts, which can reduce measurable free ITC levels, alter bioavailability, or modify biological activity [73]. Evidence from fermented broccoli products supplemented with lactoferrin indicates that added proteins can modify ITC profiles during in vitro digestion, supporting the relevance of protein–ITC interactions in complex food matrices [74]. From a nutritional and technological perspective, these findings suggest that protein-rich *Brassica*-based formulations require careful evaluation, especially when the intended outcome is predictable ITC delivery.

### 6.3. Encapsulation and Delivery Systems

Encapsulation technologies, including microencapsulation, nanoencapsulation, and liposomal delivery, offer a formulation-based strategy to improve the stability and bioavailability of ITCs. These systems can protect ITCs from degradation during storage, processing, and digestion, while also enabling more controlled release and potentially improved cellular uptake [25,77]. Encapsulated sulforaphane has been reported to show greater stability during storage and processing than free sulforaphane and may support enhanced cellular uptake and biological activity [25].

These technologies are particularly relevant for functional foods, dietary supplements, and nutraceutical formulations in which precise and reproducible ITC delivery is required. However, encapsulation should not be treated simply as a technological add-on. Its usefulness depends on whether the system improves biologically relevant exposure rather than only analytical recovery or chemical stability. Therefore, encapsulated ITC formulations should ideally be evaluated using a combination of stability testing, in vitro digestion, bioaccessibility assessment, and, where possible, biomarkers of exposure.

Collectively, matrix effects can be grouped into three functionally relevant levels. First, meal-level factors, including pH-modifying co-ingredients and buffering components, may influence myrosinase activity and hydrolysis direction, although their effects are usually weaker than those of processing intensity and tissue disruption [6,9,14]. Second, molecular interactions within the food matrix, particularly lipid partitioning and protein–ITC adduct formation, may alter the measurable free ITC fraction, stability, and bioaccessibility [72,73,74]. Third, formulation strategies such as encapsulation may improve ITC stability and controlled release, but should be evaluated using digestion-based and exposure-oriented endpoints rather than chemical retention alone [25,77]. Thus, matrix design should be interpreted as a complementary determinant of *Brassica* functionality, linking food-level ITC formation with bioaccessibility and realized exposure.

## 7. From Food Matrix to Human Exposure: Bioaccessibility, Gut Transformation, and the Limits of Prediction

### 7.1. Bioaccessibility and In Vitro Digestion

Bioaccessibility, defined as the fraction of a compound released from the food matrix and potentially available for absorption, is a critical determinant of ITC bioavailability. This distinction between release, availability, and actual uptake is central to the interpretation of glucosinolate-derived functionality, particularly because food processing strongly influences both precursor transformation and downstream bioavailability [8,78]. In vitro digestion models indicate that ITC bioaccessibility from *Brassica* foods can vary widely, ranging from approximately 10% to 80%, depending on processing, matrix composition, and digestion conditions [14,72]. Short steaming, particularly for 3–5 min, appears to support sulforaphane bioaccessibility more effectively than boiling or prolonged steaming, which reduce both enzymatic activity and the availability of bioactive hydrolysis products [14,36].

Gastric pH is another important digestive determinant of the transition from food-level ITC formation to potential absorption. Low gastrointestinal pH conditions can reduce myrosinase activity and shift glucosinolate hydrolysis toward nitrile formation [6,9]. At strongly acidic pH values, approximately 2.0–3.0, which are typical of fasting gastric conditions, plant myrosinase is largely inactivated and glucosinolates may pass unhydrolyzed into the small intestine. At postprandial pH values, approximately 4.0–5.0, myrosinase may retain partial activity, allowing some additional ITC formation during digestion [9].

### 7.2. Gut Microbiota and Colonic Transformation

When glucosinolates escape hydrolysis in the food matrix or upper gastrointestinal tract, the gut microbiota can mediate their conversion to ITCs, nitriles, and related metabolites. However, the extent and functional relevance of this process depend strongly on host-specific microbial composition and metabolic capacity [16,79,80]. Some individuals may behave as relatively efficient microbial converters, whereas others show limited conversion, which complicates dietary recommendations and intervention design because identical glucosinolate intakes may lead to markedly different ITC exposures [2,7,21]. This inter-individual variability likely reflects differences not only in overall microbiota composition but also in the abundance and metabolic activity of microbial populations capable of glucosinolate hydrolysis, the availability and chemical structure of the glucosinolate substrate, and the broader dietary context [16,19,20,79]. Accordingly, taxonomic composition alone may not fully predict functional glucosinolate-converting capacity, and microbiota-mediated ITC formation should be regarded as a variable functional phenotype rather than a uniform property of the gut microbiome.

Recent evidence further suggests that cooked broccoli may itself reshape cecal microbiota composition and influence glucoraphanin metabolism, indicating that host response and substrate conversion may be dynamically linked rather than independent processes [81]. Dmytriv et al. [19] recently emphasized that gut microbiota can convert glucoraphanin into sulforaphane and related compounds, but that this conversion is highly dependent on microbial and host-related context. Although microbial conversion may partially compensate for reduced plant myrosinase activity, it remains less predictable than hydrolysis occurring before ingestion or during the early stages of digestion. For this reason, colonic ITC formation should be regarded as a supplementary rather than primary route of bioactive delivery [7,21].

**Figure 2:**
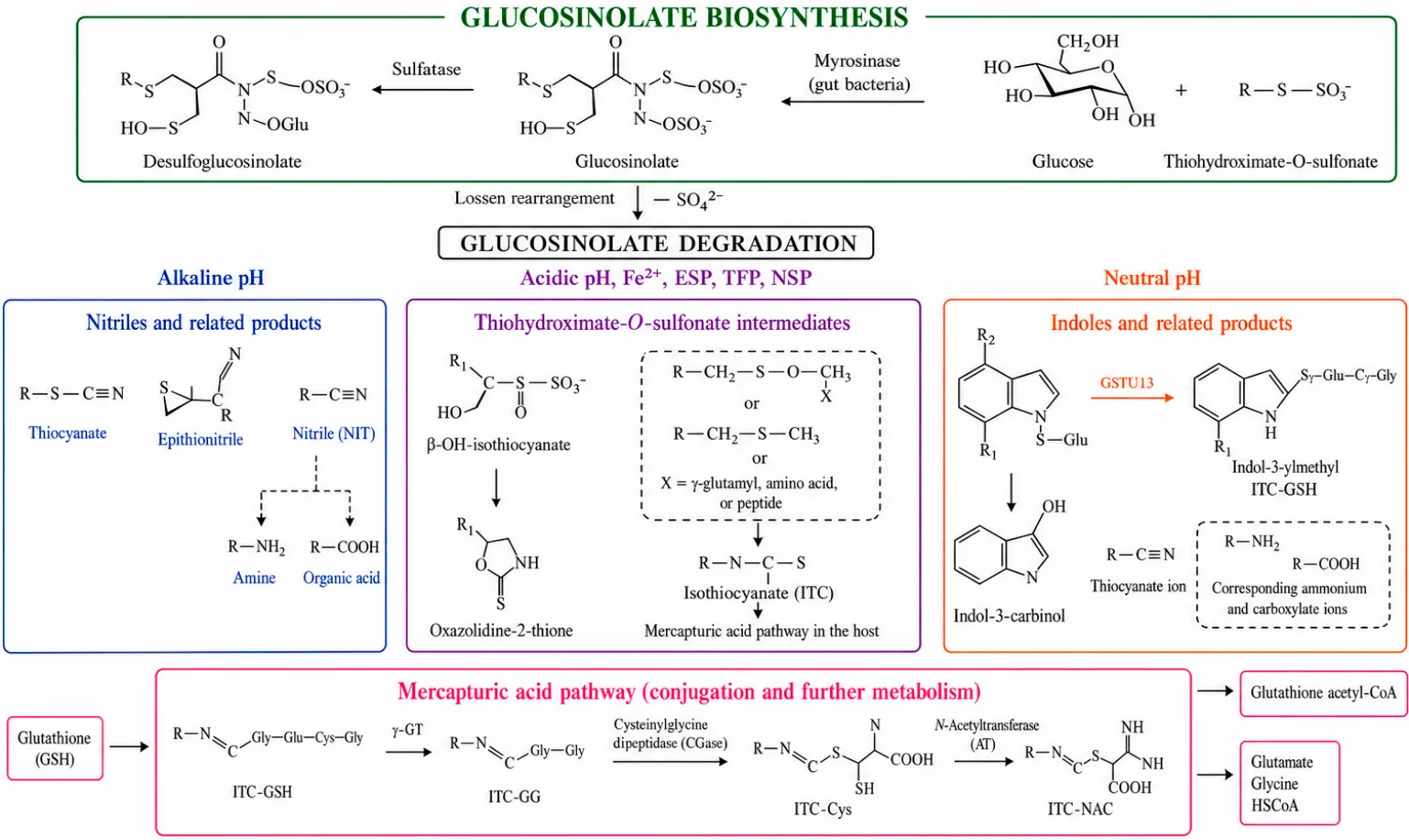
Glucosinolate degradation pathways and host metabolism of isothiocyanates. The scheme illustrates how glucosinolates can be converted under different biochemical and physicochemical conditions into isothiocyanates, nitriles, epithionitriles, thiocyanates, oxazolidine-2-thiones, and indole-derived products, and how the formed isothiocyanates are subsequently metabolized through the mercapturic acid pathway to urinary conjugates. Abbreviations: GSH, glutathione; γ-GT, γ-glutamyltransferase; CGase, cysteinylglycine dipeptidase; AT, *N*-acetyltransferase; Glu, glutamate; Gly, glycine; NIT, nitrile; ITC, isothiocyanate; NAC, *N*-acetylcysteine. Based on selected mechanisms described in Barba et al. [8], Bricker et al. [82], Castro-Torres et al. [72], Shekarri et al. [83].

To integrate these processes, Figure 2 summarizes the major degradation routes of glucosinolates and the subsequent host metabolism of ITCs through the mercapturic acid pathway.

### 7.3. Absorption, Metabolism, and Excretion

As illustrated in Figure 2, ITCs formed before ingestion, during digestion, or through microbial transformation may be absorbed and subsequently metabolized in the host. After absorption, ITCs are rapidly conjugated with glutathione via glutathione S-transferases and metabolized through the mercapturic acid pathway [73,82]. Sulforaphane metabolites, including sulforaphane-glutathione, sulforaphane-cysteine, and sulforaphane-N-acetylcysteine, are excreted in urine within approximately 8–24 h and are commonly used as biomarkers of ITC exposure [82,83]. However, urinary metabolites should be interpreted carefully. They reflect absorbed and metabolized ITCs rather than total glucosinolate content or total ITC formation in the food matrix. Some ITCs may be degraded before absorption, bound to proteins or other nucleophilic matrix components, transformed by the microbiota, or excreted through non-urinary routes [82]. Consequently, exposure biomarkers provide a stronger indication of realized bioactive delivery than precursor measurements alone, but they do not fully explain the upstream mechanisms responsible for variability in ITC formation [84]. This distinction reinforces the need to integrate food-level conversion, digestive bioaccessibility, microbial transformation, and urinary metabolite data when evaluating the nutritional functionality of *Brassica* foods.

## 8. Analytical Interpretation and the ITC-Yield Framework for Evaluating *Brassica* Foods

The preceding sections identify a sequence of analytically distinct endpoints, from precursor abundance to realized exposure. The following subsections examine how glucosinolate content, ITC formation, conversion efficiency, bioaccessibility, and exposure biomarkers should be interpreted together rather than as interchangeable measures.

### 8.1. Glucosinolate Quantification: Precise but Incomplete

Glucosinolate quantification is analytically well established and is typically performed using HPLC with UV or MS detection, often after desulfation [22]. This approach is valuable for comparing species, cultivars, tissues, developmental stages, and processing effects. However, it remains a measurement of precursor abundance rather than functional output. As emphasized by Hanschen et al. [6] and Oliviero et al. [7], glucosinolate content alone does not account for myrosinase activity, ESP activity, pH, temperature, tissue disruption, processing history, or matrix effects, all of which can strongly influence whether hydrolysis is directed toward ITCs or alternative products. This distinction is central to the interpretation of *Brassica* foods. A material with high glucosinolate content may still generate limited ITC exposure if myrosinase is inactivated, if ESP-mediated nitrile formation predominates, or if the matrix limits compound release and bioaccessibility. Conversely, a product with moderate glucosinolate content may be more functionally relevant if conversion efficiency is high. Thus, glucosinolate profiling is necessary, but insufficient: it defines the biochemical starting point, not the nutritional endpoint.

### 8.2. ITC Quantification: Challenges and Artifacts

Direct ITC quantification is closer to the functional endpoint, but it is analytically more demanding. ITCs are volatile, reactive, and chemically unstable, which makes them susceptible to degradation during sample preparation, extraction, storage, and analysis [6,25]. They may also react with proteins, amino acids, and other nucleophilic matrix components, forming adducts that are not always captured by standard analytical procedures [73]. This is particularly relevant for complex or formulated foods, where the measurable free ITC fraction may not fully represent the total ITC-related exposure potential.

Cyclocondensation methods, including 1,2-benzenedithiol derivatization, are widely used for ITC analysis, but they also have limitations, including incomplete recovery of some ITCs and possible non-specific reactions [6]. These issues complicate comparisons across studies and partly explain why precursor-based interpretation remains common despite its biological limitations. Emerging techniques may improve mechanistic understanding. For example, Shikano et al. [49] showed that imaging mass spectrometry can provide spatial information on ITC formation within plant tissues, revealing localized and heterogeneous conversion patterns that are not visible in bulk measurements. However, such approaches are not yet routine tools for food analysis. Consequently, standardized and validated methods for ITC quantification in complex *Brassica*-based matrices remain essential for reliable nutritional interpretation [6,7].

### 8.3. Conversion Efficiency as the Analytical Bridge

Conversion efficiency, understood as the proportion of glucosinolates converted into ITCs, provides the missing analytical bridge between precursor abundance and bioactive product formation. Although it is rarely reported, it is one of the most functionally relevant metrics for evaluating *Brassica* foods [30,33]. Unlike glucosinolate content alone, conversion efficiency integrates several determinants of functionality, including myrosinase activity, ESP activity, pH, temperature, tissue disruption, processing intensity, and matrix effects [6,46].

For operational purposes, and consistent with previous quantitative approaches to glucosinolate bioconversion [33,85], conversion efficiency should be expressed on a molar precursor–product basis under explicitly defined experimental conditions. For a specific glucosinolate–isothiocyanate pair, ITC conversion efficiency may be expressed as:

Conversion efficiency (%) = (molar amount of the corresponding ITC formed/ initial molar amount of the available precursor glucosinolate) × 100.

where ITCs are already present before processing or hydrolysis, baseline concentrations should be taken into account, and analytical recovery should be considered whenever possible. For samples containing multiple glucosinolates, calculation should preferentially be based on chemically assignable precursor–product pairs rather than on total glucosinolate and total ITC pools. Particular caution is required for indole glucosinolates, because their initially formed isothiocyanates are unstable and undergo rapid rearrangement to indole-derived products; therefore, a simple ITC-based conversion ratio is not directly applicable to all glucosinolate classes.

Meaningful comparison of conversion efficiency across studies additionally requires standardized reporting of the plant species and cultivar, tissue type, fresh- or dry-weight basis, degree of tissue disruption, initial glucosinolate concentration, corresponding ITC concentration, hydrolysis or processing time, temperature and pH, endogenous or exogenous myrosinase status, and the analytical method used for both precursor and product quantification [6,7,30,33]. Recovery correction and the use of appropriate internal or external standards should also be reported where applicable, particularly because ITCs are reactive and chemically unstable compounds whose measurable concentrations may be affected by sample handling, matrix interactions, and analytical conditions [6,25,72]. Importantly, food-level conversion efficiency should be distinguished from exposure-derived estimates based on urinary ITC metabolites, because the latter integrate not only glucosinolate hydrolysis but also gastrointestinal and microbial transformation, absorption, conjugation, metabolism, and excretion [81,82].

Reporting conversion efficiency alongside glucosinolate content would make analytical data more useful for breeding, food processing, product formulation, and dietary guidance [31,66,84]. Two *Brassica* samples may contain similar glucosinolate levels but differ substantially in ITC-forming potential because of differences in enzyme activity, specifier-protein activity, tissue structure, or processing history [30,62,85]. In such cases, precursor quantification may suggest comparable functional value, whereas conversion efficiency would reveal biologically relevant differences [28,53,66]. Therefore, conversion efficiency should be treated not as an optional analytical detail, but as a key interpretative metric linking plant composition with food-level ITC formation [62,66].

**Figure 3:**
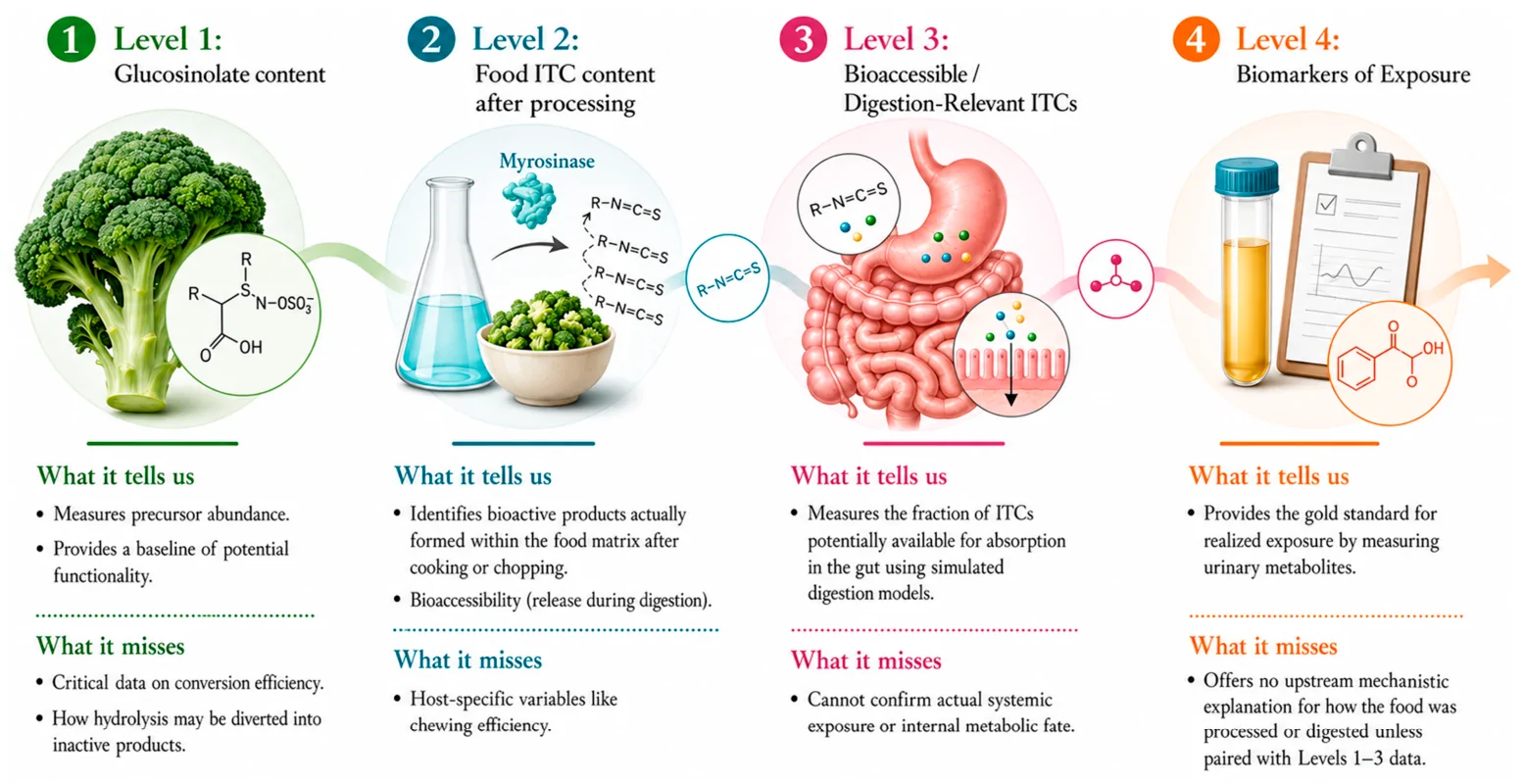
Analytical levels of evidence for *Brassica* functionality, showing why glucosinolate content alone is insufficient to infer bioactive delivery or realized exposure.

Before defining the ITC-yield framework, it is useful to distinguish the analytical levels at which *Brassica* functionality can be evaluated. These levels differ in what they reveal about biological relevance: glucosinolate content indicates precursor abundance [86,87,88]; food-level ITC content indicates products actually formed after preparation or processing [11,57,62,89]; bioaccessibility assays estimate the fraction potentially available for absorption [8,31,71,79]; and urinary metabolites provide the most direct indication of realized exposure [81,82,90,91]. This hierarchy is summarized in Figure 3.

The scheme distinguishes four complementary analytical levels: glucosinolate content, ITC formation after processing, digestion-relevant bioaccessibility, and biomarkers of exposure. Interpretative confidence increases toward exposure biomarkers, but mechanistic attribution requires integration across all levels.

### 8.4. Defining the ITC-Yield Framework

Building on the analytical hierarchy shown in Figure 3, the proposed ITC-yield framework is based on a simple premise: the health-related functionality of *Brassica* foods is determined not only by the amount of glucosinolates present in the raw material, but by the amount of bioactive ITCs that are formed, preserved, released, absorbed, and reflected in exposure biomarkers. In this framework, glucosinolate content remains an important starting point, but it should be interpreted together with ITC yield, conversion efficiency, digestive bioaccessibility, and biomarkers of exposure.

The framework rests on four principles. First, breeding strategies, processing approaches, and dietary recommendations could place greater emphasis on ITC yield alongside glucosinolate content [6,28,30]. Second, ITC yield is context-dependent and results from the interaction of plant-related factors, such as glucosinolate structure, myrosinase activity, and ESP activity; processing-related factors, such as temperature, pH, and duration; and person-related determinants, including chewing behavior, gastric pH, and gut microbiota [2,7]. Third, maximizing ITC yield requires systems-level integration of plant breeding, food processing, and consumption strategies, rather than isolated optimization of a single variable [92]. Fourth, implementation of this framework requires standardized and validated methods for ITC quantification and conversion-efficiency assessment across food and biological contexts [6,7].

### 8.5. Implementing the ITC-Yield Framework

For practical implementation, the ITC-yield framework can be organized across plant, processing, product-development, and dietary levels. At the plant level, selection should consider not only high glucosinolate content, but also high myrosinase activity, low ESP activity, favorable glucosinolate profiles, such as high glucoraphanin content, and tissue-specific distribution in edible portions [92,93]. Tissue-specific breeding may be particularly important because florets, stems, outer and inner leaves, young and mature leaves, flesh, peel, and sprouts differ substantially in glucosinolate accumulation, myrosinase activity, ESP activity, and hydrolysis-product profiles [30,37].

At the processing level, culinary and technological strategies should aim to preserve or enhance ITC formation. Such strategies include short steaming, brief microwaving, HPP at approximately 400–600 MPa, controlled fermentation at moderately acidic pH, and hydrolysis-before-cooking approaches [12,59,67]. Conversely, boiling, prolonged heating, and excessive acidification should be avoided when the aim is to maximize ITC yield, because they may inactivate myrosinase, leach glucosinolates, or shift hydrolysis toward less desirable products [6,11]. At the product-development level, processed *Brassica* foods may be improved by supplementing exogenous myrosinase, using encapsulation to stabilize ITCs, and designing matrices that favor ITC formation and bioaccessibility [13,25]. Functional foods and supplements should therefore be formulated on the basis of ITC yield and exposure potential, not only total glucosinolate content [15,21].

At the dietary-guidance level, practical recommendations include consuming raw or lightly steamed vegetables, chopping before cooking, co-consuming cooked *Brassica* foods with myrosinase-rich ingredients such as mustard or radish, and avoiding prolonged boiling when possible [32,36]. However, inter-individual variability in ITC conversion should also be considered. Differences in chewing behavior, gastric conditions, gut microbiota, and host metabolism mean that identical glucosinolate intakes may produce different ITC exposures between individuals [2,76].

Table 2 provides an illustrative comparison of tissue-specific glucosinolate content, enzymatic determinants, and estimated ITC conversion efficiency across selected *Brassica* materials.

Overall, Table 2 illustrates that tissue type may substantially influence the enzymatic determinants and estimated ITC-forming potential of *Brassica* materials. These literature-derived trends support the consideration of edible-tissue characteristics in breeding, processing, product development, and dietary evaluation, while the reported ranges should not be treated as directly comparable quantitative profiles.

## 9. Implications for Product Development, Dietary Recommendations, and Food Innovation

### 9.1. Product Development and Quality Control

The ITC-yield framework has direct implications for the development and standardization of *Brassica*-based foods, ingredients, and supplements. Instead of selecting raw materials solely on the basis of glucosinolate content, formulators should assess ITC-forming potential under realistic processing, storage, formulation, and consumption conditions [7,16]. Quality control should therefore include ITC yield or conversion efficiency as functional specifications, alongside conventional parameters such as glucosinolate content, color, texture, and sensory quality [6,25].

Functional foods and dietary supplements represent a particularly important application area. Broccoli sprout extracts, sulforaphane-enriched powders, fermented *Brassica* products, and formulated vegetable-based ingredients can be standardized to deliver more consistent ITC exposure, thereby reducing the variability inherent in whole foods [21,38]. Encapsulation technologies may further improve ITC stability, protect sensitive compounds during storage and digestion, and enable more controlled delivery formats [25,76]. However, such products should be evaluated not only for chemical stability, but also for bioaccessibility and, where possible, biomarkers of exposure.

### 9.2. Dietary Recommendations and Public Health

Current dietary guidance generally emphasizes increasing *Brassica* vegetable consumption, but often provides limited information on preparation methods that influence ITC formation and exposure [2,16]. The ITC-yield framework allows more precise and actionable recommendations, including consumption of raw or lightly steamed vegetables, chopping before cooking, avoidance of prolonged boiling, and co-consumption of cooked *Brassica* foods with myrosinase-rich ingredients such as mustard or radish [32,36]. These strategies may enhance ITC exposure without requiring an increase in total vegetable intake. Public health interventions focused on cancer prevention, cardiovascular protection, or inflammation could incorporate ITC yield alongside glucosinolate intake when evaluating *Brassica* exposure [31]. In intervention studies, urinary ITC metabolites may provide useful biomarkers of realized exposure, where appropriate, particularly when interpreted together with dietary intake, processing conditions, and food-level conversion data [81,82]. At the same time, preparation practices, matrix composition, and inter-individual variability should be documented more carefully, because they can substantially influence exposure even when intake appears similar.

### 9.3. Food Innovation and Sustainability

Despite their mechanistic appeal, not all ITC-yield-enhancing strategies are equally feasible in industrial practice. Hydrolysis-before-cooking may be effective under controlled culinary conditions, but in large-scale food processing it introduces additional residence time, complicates workflow design, and may increase microbiological risk unless carefully managed [7,12]. Similarly, exogenous myrosinase supplementation offers a promising route to restore ITC formation in processed foods, but its application must be evaluated in relation to ingredient cost, processing stability, regulatory constraints, and potential changes in sensory profile, particularly pungency [13,15,101]. Successful industrial translation will therefore require technologies that balance biochemical effectiveness with process safety, economic viability, sensory quality, and consumer acceptability.

The ITC-yield framework also creates opportunities for sustainable food innovation. *Brassica* by-products, including stems, leaves, and peels, often contain valuable glucosinolate pools but are discarded or underused because of low palatability or limited technological application. Processing these by-products to improve ITC yield through fermentation, HPP, or enzyme supplementation could support the development of value-added ingredients for functional foods, nutraceuticals, or animal feed [102,103].

Fermented *Brassica* products, such as kimchi and sauerkraut, are culturally important and widely consumed, yet their ITC content and conversion efficiency remain insufficiently characterized [40,68]. Optimizing fermentation conditions to enhance ITC yield while maintaining sensory quality and microbial safety may improve the functional value of these traditional foods [38,69]. This is particularly relevant for product categories in which consumers already accept fermentation as part of the sensory and nutritional identity of the food.

## 10. Limitations of the ITC-Yield Framework and Future Research Priorities

However, the proposed ITC-yield framework should currently be regarded as a conceptual and interpretative model rather than as a prospectively validated predictive tool. Food-level ITC yield is not equivalent to digestive bioaccessibility, systemic bioavailability, realized exposure, or biological effectiveness, each of which is additionally shaped by gastrointestinal transformation, absorption, metabolism, excretion, dose, and host responsiveness [2,7,16,81,82]. The framework may also simplify compound-specific differences among individual ITCs and should not imply that all health-related properties of *Brassica* foods are mediated exclusively through ITC formation. Non-ITC hydrolysis products, particularly indole-derived compounds, may also contribute to biological activity and require separate consideration [2,16]. Moreover, quantitative interpretation within the framework depends on how the available precursor pool is defined, when samples are collected relative to tissue disruption, processing, digestion, or metabolism, and which analytical procedures are used for glucosinolate and ITC quantification [6,7]. Accordingly, the framework should complement, rather than replace, glucosinolate profiling, bioaccessibility assessment, and exposure-biomarker measurements. Its predictive and translational value will ultimately require prospective validation across different *Brassica* species, cultivars, tissues, processing systems, dietary contexts, and human populations.

Beyond these conceptual limitations, several methodological and evidential challenges restrict the practical implementation of an ITC-yield-centered framework for evaluating *Brassica* functionality. One of the most important challenges concerns analytical standardization. As emphasized by Hanschen et al. [6], ITC quantification is complicated by volatility, chemical reactivity, instability, and matrix-dependent analytical artifacts, which continue to limit comparability between studies. Therefore, standardized and inter-laboratory validated methods for ITC quantification in complex food matrices remain urgently needed, particularly because processing conditions strongly affect both glucosinolate transformation and measurable ITC formation [7]. In parallel, comprehensive databases of conversion efficiency across *Brassica* species, cultivars, tissues, processing methods, and formulation systems would provide a stronger basis for evidence-based recommendations and predictive modeling [30,33].

A further limitation concerns inter-individual variability. The roles of genetics, gut microbiota, habitual diet, gastrointestinal conditions, and health status in shaping ITC conversion and exposure remain insufficiently resolved, although they are central to the future development of personalized nutritional strategies [2,78]. In parallel, ITC stability during storage, distribution, and shelf-life requires more systematic investigation, particularly in processed foods, ready-to-eat products, and supplement systems. Although formulation and delivery systems may improve stability and bioavailability, degradation during storage or processing may still substantially alter realized exposure [25,104]. Stronger dose–response data are also needed to link ITC exposure with specific health outcomes, including cancer prevention, inflammatory status, and cardiovascular protection. Angelino and Jeffery [31] provided an important mechanistic basis for interpreting the biological activity of ITCs, but stronger human evidence is still required to translate these mechanisms into robust dietary guidance. Future studies should therefore combine controlled dietary interventions with well-characterized *Brassica* foods, standardized analytical methods, and exposure biomarkers. Another important research direction concerns tissue-specific breeding strategies aimed at increasing myrosinase activity and reducing ESP activity in edible tissues, while maintaining agronomic performance, sensory quality, and consumer acceptability [37,92].

Human intervention studies provide important evidence that enhanced glucosinolate hydrolysis can increase systemic ITC exposure, but this should not be equated with demonstrated long-term health benefit [83]. Controlled studies have shown that the presence of active or exogenous myrosinase can substantially increase sulforaphane bioavailability, while recent human feeding data also demonstrate pronounced inter-individual variability in sulforaphane absorption, metabolism, and excretion [6,15,20,21,90,91]. Such variability appears to reflect differences in gastrointestinal conditions, microbial metabolism, and host handling of ITCs, reinforcing the distinction between food-level conversion efficiency and realized systemic exposure. Importantly, urinary ITC metabolites are valuable exposure biomarkers, but they integrate multiple downstream processes, including hydrolysis, absorption, conjugation, metabolism, and excretion, and therefore cannot be interpreted as direct measures of food-level ITC yield or biological effectiveness. Moreover, robust dose–response relationships between quantitatively defined ITC exposure and long-term clinical outcomes remain insufficiently established. Future intervention studies should therefore characterize precursor dose, food-level ITC formation or conversion efficiency, exposure biomarkers, and clinically relevant endpoints in parallel.

Emerging processing technologies, including pulsed electric fields, ultrasound, and cold plasma, also deserve further evaluation as tools for enhancing ITC formation while preserving sensory quality and product acceptability [66,102]. More broadly, both thermal and non-thermal processing can substantially modify glucosinolate retention and the formation of their bioactive derivatives, emphasizing the need to evaluate processing technologies in terms of functional output rather than precursor preservation alone [105]. Finally, strategies for modulating the gut microbiota to improve in vivo ITC conversion, including prebiotics, probiotics, and broader dietary interventions, represent an important frontier in the context of personalized nutrition. This direction is particularly relevant because microbiota-mediated conversion may partly compensate for reduced plant myrosinase activity, but remains highly variable and difficult to predict at the individual level [78,79].

Taken together, four research priorities should guide the further development of the ITC-yield framework: (i) interlaboratory standardization of ITC quantification and conversion-efficiency reporting [6,7]; (ii) prospective validation across *Brassica* species, cultivars, tissues, processing conditions, and formulation systems [30,33]; (iii) controlled human studies linking precursor dose and food-level ITC formation with exposure biomarkers and clinically relevant outcomes [81,82,83]; and (iv) predictive models integrating matrix composition, processing history, gastrointestinal conditions, and microbiota-dependent variability [2,78,79]. Addressing these priorities will determine whether the framework can progress from a conceptual model to a reproducible and translationally useful tool for food design, dietary guidance, and intervention research.

## 11. Conclusions

The functional value of *Brassica* vegetables is not adequately captured by glucosinolate content alone. Although glucosinolates are essential precursors, their nutritional relevance depends on their conversion into bioactive isothiocyanates, a process shaped by myrosinase activity, specifier proteins, processing conditions, food matrix effects, gastrointestinal transformation, and inter-individual variability. The evidence synthesized in this review supports an ITC-yield-centered interpretation of *Brassica* functionality, in which conversion efficiency characterizes food-level ITC formation, whereas subsequent bioaccessibility and host-related factors determine realized exposure. Considered together, these complementary levels may provide a more biologically meaningful basis for breeding, food processing, product development, dietary guidance, and intervention studies.

Future research would benefit from greater standardization of ITC quantification, broader reporting of conversion efficiency, and parallel assessment of exposure biomarkers in human studies. Within the proposed framework, ITC yield may serve as a useful food-level functional metric for evaluating the bioactive potential of *Brassica* foods, but it should not be interpreted as a direct surrogate for systemic bioavailability or biological effectiveness.

## Figures and Tables

**Table 1:** Illustrative literature-derived ranges for the effects of processing methods on glucosinolate-to-isothiocyanate conversion and related enzymatic determinants.

Processing Method	Temperature/Conditions	Myrosinase Activity (% Retained)	ESP Activity (% Retained)	Estimated ITC Conversion Efficiency (%)	Key Mechanistic Insight	Selected References
Raw (uncooked)	Ambient	100	100	40–70	Baseline conversion depends on tissue disruption, endogenous enzyme activity, and matrix characteristics.	[52,53]
Boiling (5–10 min)	100 °C	<10	<10	<5	Rapid myrosinase inactivation and glucosinolate leaching severely limit ITC formation.	[11,53,54]
Steaming (3–5 min)	80–90 °C	60–80	40–60	50–80	Short steaming partly preserves myrosinase while improving enzyme–substrate contact.	[11,54,55]
Steaming (>10 min)	80–90 °C	10–30	10–30	10–30	Prolonged steaming progressively reduces enzymatic conversion efficiency.	[11,54,55]
Microwaving (1–2 min)	Variable	70–90	50–70	60–85	Short microwave treatment may enhance ITC formation through tissue disruption with limited enzyme loss.	[50,54,56]
Microwaving (>3 min)	Variable	20–40	20–40	15–35	Longer exposure increasingly compromises myrosinase stability and ITC yield.	[50,53,56]
Stir-frying (<3 min)	150–200 °C	50–70	30–50	45–70	Brief high-temperature treatment may retain partial hydrolytic capacity, especially after pre-hydrolysis.	[12,57,58]
Stir-frying (>5 min)	150–200 °C	10–30	10–30	10–25	Extended stir-frying markedly reduces enzymatic conversion.	[53,57,58]
High-pressure processing (400–600 MPa)	Ambient	80–100	20–40	70–90	Moderate pressure may suppress ESP while preserving myrosinase, favoring ITC formation.	[59,60]
High-pressure processing (>600 MPa)	Ambient	30–50	<10	30–50	Excessive pressure increasingly destabilizes myrosinase and lowers conversion efficiency.	[59,60]
Fermentation (mild acidification; pH 5.0–5.5)	20–30 °C	60–80	40–60	50–75	Mild acidification may sustain partial conversion, depending on microbial and matrix conditions.	[53,61]
Fermentation (strong acidification; pH <4.5)	20–30 °C	20–40	60–80	15–35	Strong acidification may reduce ITC formation and promote alternative hydrolysis products.	[29,61,62]
Hydrolysis-before-cooking	Ambient + cooking	0 (post-cooking)	0 (post-cooking)	70–95	Pre-hydrolysis enables ITC formation before thermal enzyme inactivation.	[12,63,64]
Exogenous myrosinase	Post-cooking	100 (added)	0 (inactivated)	60–85	External myrosinase sources can restore post-cooking glucosinolate hydrolysis.	[7,13,18,65]

Note: The numerical ranges presented in Table 1 are literature-derived estimates synthesized from the experimental findings reported in the studies cited for each processing condition. They are intended to illustrate the approximate magnitude and direction of processing effects rather than to represent pooled estimates, meta-analytic ranges, or universal reference values. Individual studies differed substantially in *Brassica* species and cultivar, tissue type, degree of tissue disruption, initial glucosinolate and enzyme status, processing time and intensity, pH, matrix composition, and analytical methodology; moreover, myrosinase activity, ESP activity, and ITC conversion were not always measured simultaneously within the same experimental system. The ranges should therefore be interpreted as comparative, mechanistically oriented approximations rather than fixed quantitative thresholds. Selected studies supporting each processing category are provided in the final column.

**Table 2:** Illustrative literature-derived ranges for tissue-specific glucosinolate content, enzymatic determinants, and estimated ITC conversion efficiency across selected *Brassica* materials.

Species/Cultivar	Tissue Type	Glucosinolate Content (μmol/g DW)	Myrosinase Activity (units/g FW)	ESP Activity (Relative Units)	Estimated ITC Conversion Efficiency (%)	Selected References
Broccoli	Florets	15–25	8–12	1–2	60–75	[12,18,37,48,88,93]
Broccoli	Stems	8–15	3–6	2–4	35–50	[12,14,18,37,48,88]
Cabbage	Leaves (outer)	10–18	5–9	3–5	40–60	[29,40,43,54,94]
Cabbage	Leaves (inner)	12–20	6–10	2–4	50–70	[29,40,43,54,94,95]
Kale	Leaves (young)	20–35	10–15	1–3	65–80	[4,23,37,46,83,96,97]
Kale	Leaves (mature)	15–25	5–8	3–6	40–55	[4,23,37,46,85,97]
Kohlrabi	Flesh	8–12	4–7	2–3	50–65	[30,33,46,47,98]
Kohlrabi	Peel	12–18	6–9	8–12	20–35	[30,43,45,46,98]
Chinese kale	Sprouts	30–50	15–20	1–2	75–90	[23,44,46,93,99,100]
Chinese kale	Mature leaves	18–28	7–11	3–5	50–65	[23,37,44,92,93,99,100]

Note: Values are illustrative literature-derived ranges synthesized from the studies cited for each material. Parameters presented within a single row were not necessarily measured simultaneously in the same study or experimental system and should not be interpreted as pooled estimates, directly comparable measurements, or fixed reference values. Actual values depend on plant material, developmental stage, processing and sample-handling conditions, matrix effects, and analytical methodology.

## Data Availability

No new data were created or analyzed in this study. Data sharing is not applicable to this article, as this is a review based on previously published literature.
